# A general Bayesian algorithm for the autonomous alignment of beamlines

**DOI:** 10.1107/S1600577524008993

**Published:** 2024-10-28

**Authors:** Thomas W. Morris, Max Rakitin, Yonghua Du, Mikhail Fedurin, Abigail C. Giles, Denis Leshchev, William H. Li, Brianna Romasky, Eli Stavitski, Andrew L. Walter, Paul Moeller, Boaz Nash, Antoine Islegen-Wojdyla

**Affiliations:** a Brookhaven National Laboratory Upton NY11973 USA; b Lawrence Berkeley National Laboratory, Berkeley, CA94720, USA; c Stony Brook University, New York, NY11974, USA; d RadiaSoft LLC, Boulder, CO80301, USA; Paul Scherrer Institut, Switzerland

**Keywords:** Bayesian optimization, automated alignment, synchrotron radiation, digital twins, machine learning

## Abstract

Autonomous methods to align beamlines can decrease the amount of time spent on diagnostics, and also uncover better global optima leading to better beam quality. The alignment of these beamlines is a high-dimensional expensive-to-sample optimization problem involving the simultaneous treatment of many optical elements with correlated and nonlinear dynamics. Bayesian optimization is a strategy of efficient global optimization that has proved successful in similar regimes in a wide variety of beamline alignment applications, though it has typically been implemented for particular beamlines and optimization tasks. In this paper, we present a basic formulation of Bayesian inference and Gaussian process models as they relate to multi-objective Bayesian optimization, as well as the practical challenges presented by beamline alignment. We show that the same general implementation of Bayesian optimization with special consideration for beamline alignment can quickly learn the dynamics of particular beamlines in an online fashion through hyperparameter fitting with no prior information. We present the implementation of a concise software framework for beamline alignment and test it on four different optimization problems for experiments on X-ray beamlines at the National Synchrotron Light Source II and the Advanced Light Source, and an electron beam at the Accelerator Test Facility, along with benchmarking on a simulated digital twin. We discuss new applications of the framework, and the potential for a unified approach to beamline alignment at synchrotron facilities.

## Abstract 2

Automated alignment methods have the potential to accelerate greatly the efficiency of experiments done at synchrotron facilities and are an important step towards fully autonomous beamlines. Presented here is a general implementation of Bayesian optimization that performs well on many beamlines across different facilities.

## 1. Introduction

Synchrotron light sources are invaluable scientific tools that allow the probing of materials across bulk, micrometre and nanometre scales. These facilities perform a wide variety of research, with applications in the study of catalysis, biological function and materials science. Several next-generation synchrotron and free-electron laser facilities are scheduled to receive upgrades which will increase their brilliance by several orders of magnitude (Borland & Blednykh, 2018 ▸; Chenevier & Joly, 2018 ▸; Galayda, 2018 ▸; White *et al.*, 2019 ▸). However, more advanced experiments will require more precise and complex optical setups.

Beamlines consist of a large number of optical components (*e.g.* mirrors, magnets, apertures), each with many degrees of freedom (corresponding to *e.g.* motors that translate, rotate and bend the components; see, for example, Fig. 4). These degrees of freedom can be highly correlated or degenerate, making beamline alignment in essence a high-dimensional (*D* ≳ 10) and highly nonlinear optimization problem.

This is typically done manually, and the design of optical systems is typically done to separate some of these dimensions and make manual alignment more feasible, *e.g.* by prefocusing and refocusing with a secondary-source aperture and a pair of Kirkpatrick–Baez mirrors. Nevertheless, as the complexity and precision of beamlines grow, the development of efficient and robust automated alignment methods is necessary for the efficient operation of light sources now and in the future. Such methods allow us to reach an acceptable level of alignment more quickly and robustly than with manual methods when realignment is necessary, saving preparation and commissioning time which could be used for experiments. They further allow us potentially to find better global optima than an operator could discover manually by considering all dimensions of the beamline simultaneously. They also represent the first step toward a fully autonomous beamline (Maffettone *et al.*, 2023 ▸).

Some attempts at beamline alignment apply methods like genetic and differential evolution (Xi *et al.*, 2015 ▸, 2017 ▸; Rakitin *et al.*, 2020 ▸; Zhang *et al.*, 2023 ▸), attempt to match beamline data to an online model (Nash, Abell, Nagler *et al.*, 2022 ▸; Nash, Abell, Keilman *et al.*, 2022 ▸) or use families of commonly used optimization algorithms (Breckling *et al.*, 2022 ▸; Morris *et al.*, 2022 ▸). These approaches are limited in that they give no guarantee of convergence to a global optimum. They also make no consideration of minimizing the number of function evaluations, and beamline optimization almost universally involves a prohibitively expensive-to-sample function, both on the real beamline (relying on the movement of precise motors, which can be slow) and on simulated digital twins [relying on computationally intensive ray-tracing (Sanchez del Rio *et al.*, 2011 ▸) or Fourier-based methods (Chubar *et al.*, 2013 ▸)], meaning that their use is intractable for large numbers of dimensions.

In contrast to the classical methods above, algorithms based on machine learning construct and fit a model to understand the effects of changing the parameter inputs, as well as the interaction of the output beam qualities (*e.g.* flux, spatial resolution, energy resolution, polarization, coherence), leading to a more efficient search of the parameter space. Some machine learning methods like reinforcement learning (Velotti *et al.*, 2022 ▸) suffer from similar drawbacks to the methods above in that they may take too long to learn enough to be useful, by which point beamline parameters and hyperparameters may have drifted substantially. From a practical point of view, then, we should greatly prefer alignment methods that converge as quickly as possible and rely on little to no prior input.

A machine learning framework well suited for expensive-to-sample functions is Bayesian optimization, which performs well with no prior information on optimization problems that are expensive-to-sample, high-dimensional and potentially very noisy. Bayesian optimization has been applied in a wide variety of contexts such as synchrotron light sources (Rebuffi *et al.*, 2023 ▸; Morris *et al.*, 2023 ▸), free-electron lasers (Duris *et al.*, 2020 ▸), particle colliders (Cisbani *et al.*, 2020 ▸) and laser plasma-based ion sources (Dolier *et al.*, 2022 ▸). These implementations, however, are typically applicable to single experiments; indeed, much of the difficulty in implementing machine learning solutions to any problem is the trade off of specificity and generality, where an algorithm that is specific enough to be effective in some context is too specific to be applied generally.

Bayesian optimization is highly generalizable in the choice of the kernel model used to describe the parameter space, and the fact that many facilities are moving toward shared software environments and shared data acquisition protocols like *Bluesky* (Allan *et al.*, 2019 ▸; Rakitin *et al.*, 2022 ▸) suggests the benefit of a general agent. This paper demonstrates an implementation of a Bayesian agent that can learn the dynamics and idiosyncrasies of a particular beamline and can thus be deployed across many different beamlines with relatively little implementation cost by applying the same code to a range of optimization problems at different synchrotron and non-synchrotron facilities.

In Sections 2, 3 and 4 we present a general but brief formulation of multi-objective Bayesian optimization with Gaussian process regression as it relates to this work [for a more thorough introduction see Frazier (2018 ▸)]. Section 5 addresses beamline-specific considerations for Bayesian optimization and Section 6 presents their implementation in a software package. Section 7 describes the application of the code on beamlines across the National Synchrotron Light Source II and Accelerator Test Facility at Brookhaven National Laboratory and the Advanced Light Source at Lawrence Berkeley National Laboratory, and presents the results of benchmarking on a simulated digital twin. Finally, Section 8 discusses the future development of the algorithm.

## 2. Bayesian optimization

Consider an expensive-to-sample black-box function *f*(*x*) with *d*-dimensional inputs 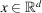. In finding the right input *x* to achieve the maximal value of *f*(*x*), it is untenable to utilize optimization methods that rely on lots of function samples. We can address this by treating the function as a stochastic process (which describes a distribution over all possible realizations of the function) and using Bayesian inference to construct a posterior distribution *p*(*f*), *i.e.* describing how likely it is that every possible function *f* is the true function.^1^ If we sample the function at points *x* = {*x*_1_, *x*_2_, …, *x*_*n*_} and observe values *y* = {*f*(*x*_1_), *f*(*x*_2_), …, *f*(*x*_*n*_)}, then we can use Bayesian inference to write our posterior belief about *f* given that we observe *x* and *y* as 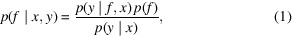
where the quantity *p*(*y* ∣ *f*, *x*) (called the *likelihood*) is the probability of observing values *y* at inputs *x* for a given function *f*, the quantity *p*(*f*) (called the *prior*) is our knowledge about the probability of a given function *f* before we have seen any data, and the quantity *p*(*y* ∣ *x*) (called the *marginal likelihood*), represents the distribution of *y* after marginalizing over the distribution of *f*. We know that the marginal likelihood must be 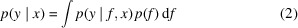
because the sum of all the probabilities in the posterior must sum to unity.

**Figure 1:**
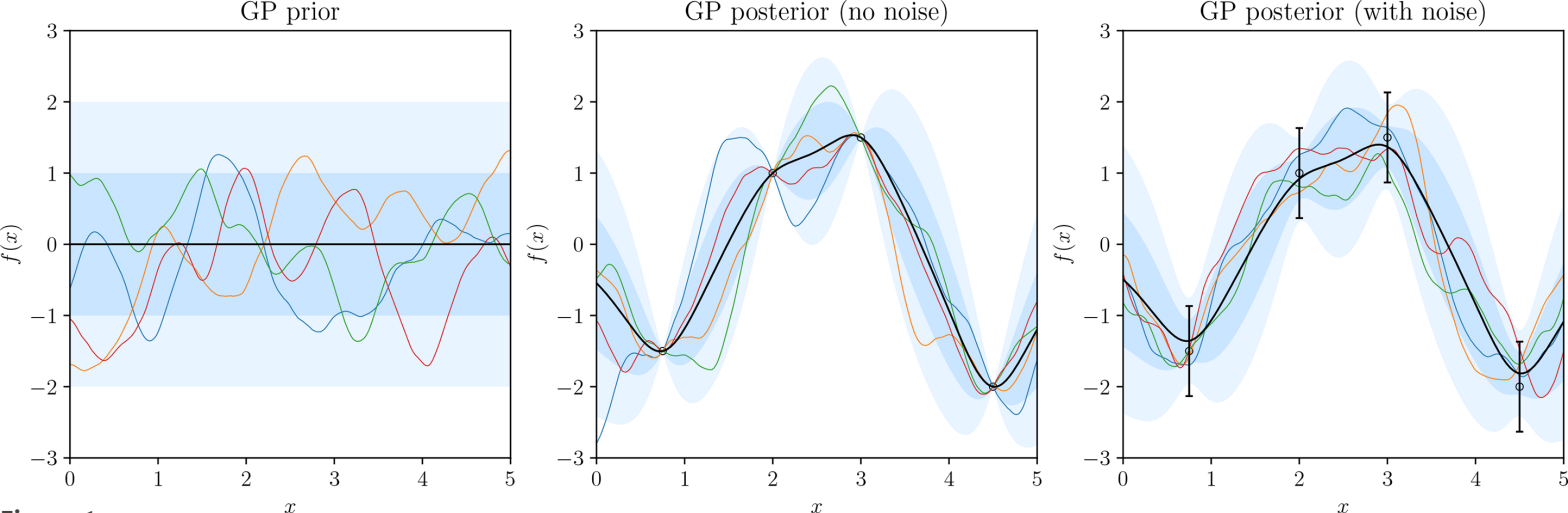
The prior distribution, noiseless posterior distribution and noisy posterior distributions for a GP with covariance 〈*f*(*x*_*i*_)*f*(*x*_*j*_)〉 = *M*_5/2_(|*x*_*i*_ − *x*_*j*_|/4), where *M*_ν_(*r*) is the Matérn function as defined in equation (19). For each distribution, we draw four random functions (colored lines). The black line represents the mean of each distribution, while the dark- and light-shaded regions represent the 1σ and 2σ intervals, respectively.

A representation of conditioning a prior on observations to construct a posterior is shown in Fig. 1 ▸. Each iteration of Bayesian optimization then consists of three steps:

(i) Estimate the posterior *p*(*f* ∣ *y*, *x*) from some historical observations (*x*, *y*).

(ii) Use the posterior to find the most desirable point 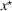 within some predefined bounds.

(iii) Sample that point and add it to our historical observations.

**Figure 2:**
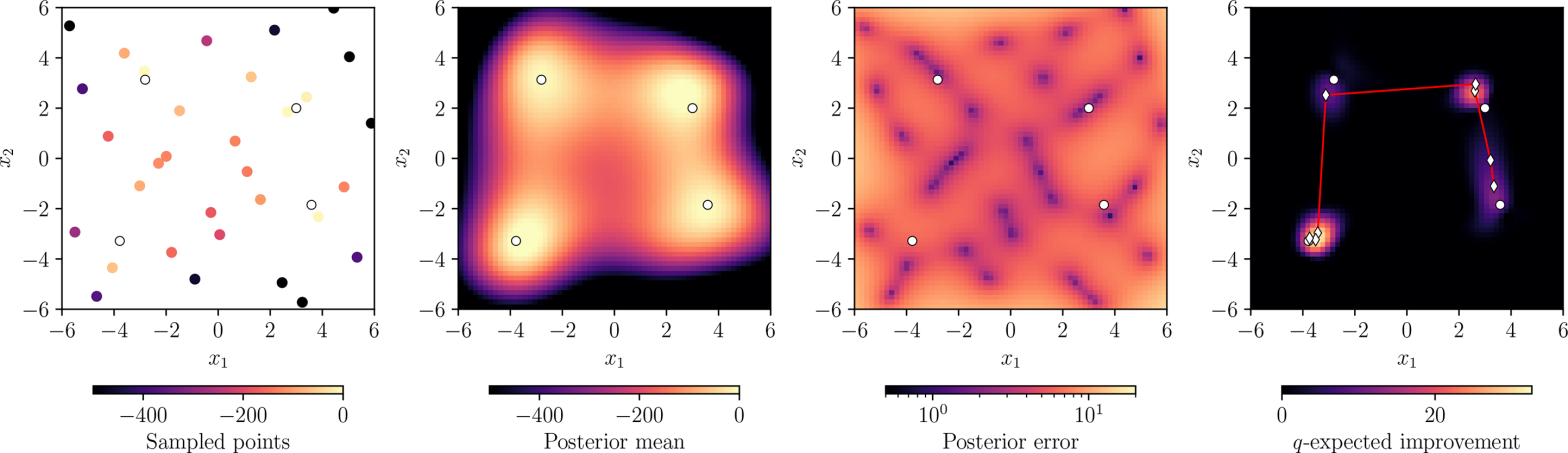
An example of an iteration of a Bayesian optimization algorithm trying to maximize the negated Himmelblau function *f*(*x*_1_, *x*_2_) = 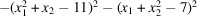, whose true global optima are marked as white circles. Using existing data points (far left) and the assumption that the function is distributed as a GP, we can use Bayesian inference to compute a posterior consisting of a mean (center left) and error (center right), upon which we can compute an acquisition function (far right) which informs us of the best points to sample. The black-edged diamonds superimposed on the acquisition function show the best eight points to sample, optimized in parallel and with the optimal routing represented by the red line.

Constructing a posterior from observations in the first step is almost always done with a Gaussian process (GP), the particulars of which are described in Section 3. Quantifying the desirability of candidate points in the second step is done using acquisition functions which are described in Section 4. A concrete example of an iteration of Bayesian optimization as applied to minimizing the Himmelblau function is shown in Fig. 2 ▸, using a GP model and an acquisition function that computes the expected improvement in the cumulative maximum by sampling each candidate point.

## 3. Gaussian process models

A GP is a stochastic process where every collection of variables *y* has a multivariate normal distribution; for notational simplicity and without loss of generality, we assume throughout this paper that all of our processes are zero mean. The GP is described entirely by the covariance matrix Σ describing the observations *y*. A GP model consists of assigning a covariance matrix to a set of sample data *y* at inputs *x* and computing the posterior mean and posterior variance at every other input. In practice, the covariance of the process is not known *a priori* and is approximated by constructing and fitting a kernel.

### 3.1. Kernels and hyperparameter optimization

We model the covariance matrix with a kernel matrix *K*(*x*, *x*′, θ), where 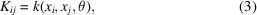
where *k* is a kernel function, *x*_*i*_ and *x*_*j*_ are two inputs, and θ is a set of hyperparameters which tune *k*. The only constraint on a kernel matrix *K* is that it is positive definite (*i.e.* it is a symmetric matrix whose eigenvalues are all strictly positive). A simplifying assumption is to require that the kernel is stationary, that is, that the correlation of the function at two inputs depends only on their distance, 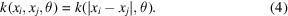
To construct our kernel, we take the hyperparameters which maximize the marginal likelihood 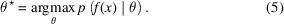
For a GP, the marginal likelihood is given by 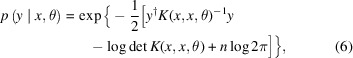

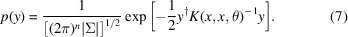


### 3.2. Posterior estimation

Once we have our kernel *K*(*x*_*i*_, *x*_*j*_, θ) and optimized hyperparameters 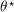, we can use GP regression to construct posteriors. Given our measurements *y* at points *x*, our posterior estimate of the distribution of the process at points 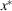 is a Gaussian distribution with posterior mean 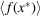 = *Af*(*x*) and posterior covariance 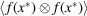 = *B*, where 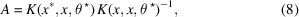

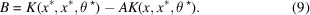
The vector of variances for each individual point is the diagonal of the posterior covariance *B*.

### 3.3. Noisy models

It may be the case that our observations are noisy, *i.e.* that observing the function at points *x* will yield *y* = *f*(*x*) + ε where ε is a random noise term. If we assume that ε is homoskedastic and Gaussian, then we can account for the noise by adding a constant noise variance σ^2^ to the diagonal of the kernel *K*. A small noise level (or ‘jitter’) is desirable even for noiseless GP models to make the Cholesky decomposition of the kernel a well conditioned problem.

## 4. Acquisition functions

The acquisition function 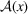 is a model of a given objective over possible inputs which, given a posterior *p*(*f* ∣ *x*, *y*), quantifies the desirability of sampling a given input *x*. For each iteration of the optimization, we optimize the acquisition function over the inputs as 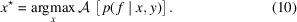
Acquisition functions over posteriors are typically cheap to compute and so classical algorithms (like LM-BFGS) are used to optimize them. (In regimes involving large volumes of data, however, computing and optimizing acquisition functions in parallel can be computationally expensive. We note the benefits of GPU-accelerated acquisition function optimization, though we do not implement it in this work.) Acquisition functions can be either analytic or non-analytic; below, we show benefits and examples of either approach.

### 4.1. Analytic acquisition functions

Analytic acquisition functions are directly computable from the posterior; as the posterior for a GP is determined entirely by the mean μ and variance σ, they may be expressed as 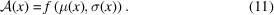
The simplest example is the expected mean 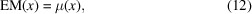
where on every iteration the algorithm will sample the point with the largest expected mean. A less risk-averse example is the expected improvement 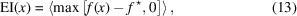
which is our expectation for how much the cumulative maximum 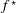 will increase if we were to sample *x*. We can compute this directly as 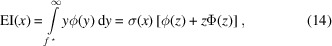
where *z* = [μ(*x*) − 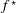]/σ(*x*), and ϕ(*z*) and Φ(*z*) are the probability density function and cumulative distribution function, respectively, of the standard normal distribution. Because repeated sampling of a point will strictly decrease the posterior variance, this algorithm will (for well behaved problems) eventually explore every point in the parameter space.

### 4.2. Monte Carlo acquisition functions

Some useful acquisition functions cannot be computed directly from the mean and variance of the posterior. Acquisition functions that involve sampling from the posterior to estimate some ensemble are more flexible and often more robust. One example of this is in selecting multiple points, as in when we want to find the best *n* points to sample given some analytic acquisition function 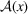: presumably they should be spread out to better cover the parameter space, but there is no obvious way to quantify and thus compute that analytically. We address this interdependence with a Monte Carlo acquisition function, where we might evaluate the acquisition of some collection of points by sampling from the posterior and taking an ensemble average of the result. There is a large benefit in sampling multiple points at once for beamline optimization, as it allows us to find a batch of points to sample and then optimally route the beamline parameters between them to reduce travel time.

Note that all analytic acquisition functions have a Monte Carlo equivalent; an example is shown in the far right-hand panel of Fig. 2 ▸, where we use the *q*-expected improvement (*q* refers to the *q*-batching formalism used to denote the axis of Monte Carlo samples) as an acquisition function to find a parallel set of eight inputs to sample next. Monte Carlo methods also allow for more sophisticated information theory-based acquisition functions like predictive entropy search (Hernández-Lobato *et al.*, 2014 ▸), maximum entropy search (Wang & Jegelka, 2017 ▸) or joint entropy search (Hvarfner *et al.*, 2022 ▸).

### 4.3. Multi-objective optimization

Optimization problems often require managing trade offs. For example, a common beamline design consists of a secondary-source aperture (SSA), which cuts off some flux in the interest of having a smaller and tighter beam. One method of multi-objective optimization is scalarization, which uses a function that maps a vector output to a scalar output, leading to one quantity to be maximized. In this work, we use affine scalarizations (*i.e.* assigning a weight to each objective and summing them) to construct a single fitness function over which to optimize an acquisition function.

There are, however, are other useful ways to carry out multi-objective optimization, such as Pareto efficient searches (*i.e.* finding the set of inputs where no one objective can be increased without decreasing some other objective). But while fully multi-objective methods do allow for more flexibility in the alignment, that flexibility is not compatible with an autonomous beamline, which must decide on a single best beam and thus must collapse the beam to a single fitness function.

## 5. Beamline-specific considerations

In this section, we look at beamline-specific considerations that improve the practical application of Bayesian optimization to the automated alignment problem. In this paper, we consider the common optimization problem of maximizing the beam power density, defined as 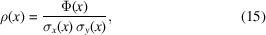
where *x* represents the beamline inputs to optimize and where Φ(*x*), σ_*x*_(*x*) and σ_*y*_(*x*) are the input-dependent flux, horizontal spread and vertical spread of the beam, respectively. These parameters are inferred from an image of the beam profile, taken using either an area detector (*e.g.* Figs. 6 and 8) or a beam stop and microscope (*e.g.* Figs. 5 and 9). In practice, it is better to model the fitness as 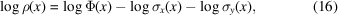
because the distribution in variations in the beam flux and size are both roughly log-normal and so their logarithms are better described by a GP. It also preserves the convexity of the problem and, being inherently dimensionless, allows us to scalarize affinely many simultaneous objectives as a single GP.

### 5.1. A kernel for latent beamline dimensions

**Figure 3:**
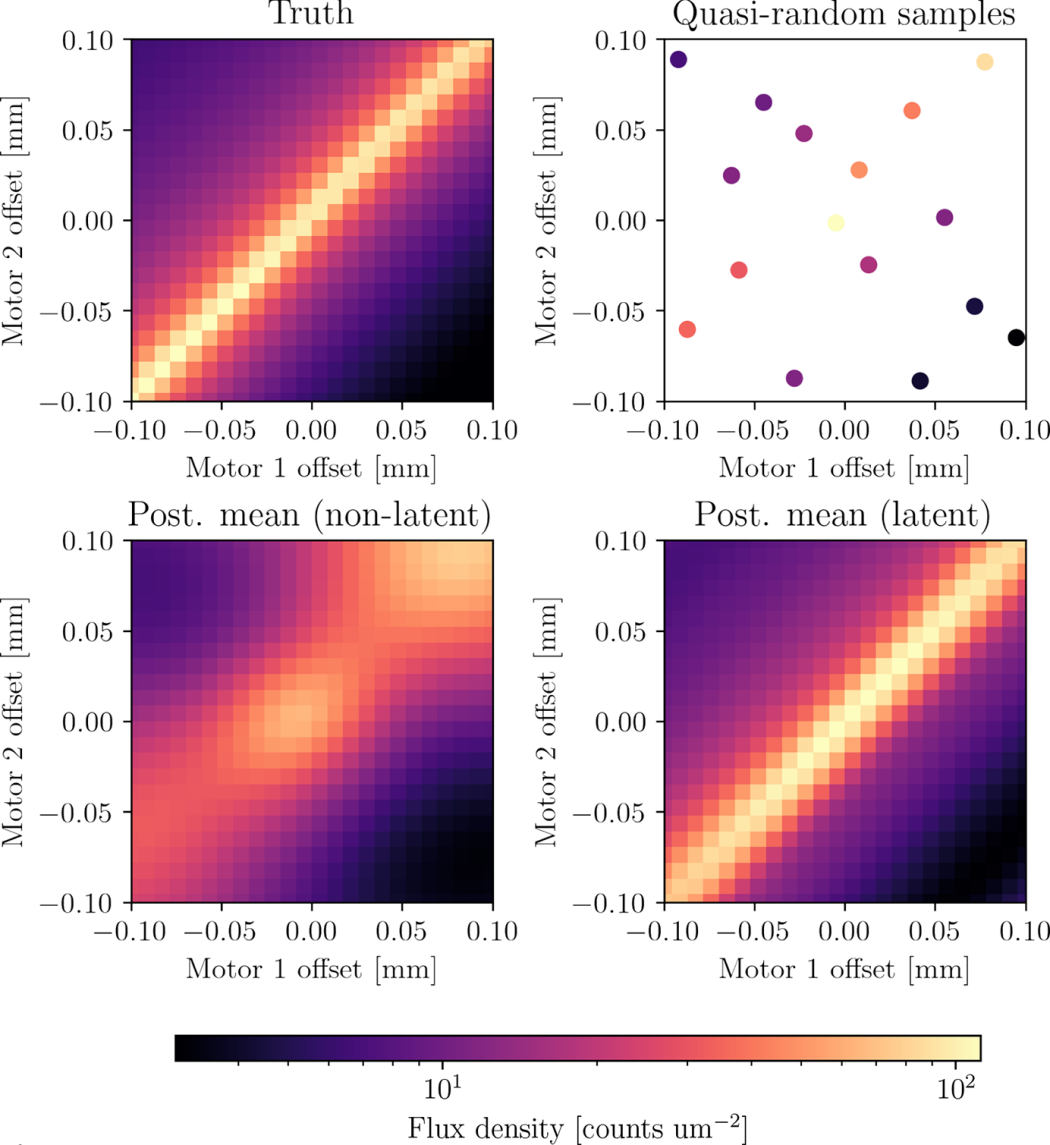
(Upper left) The result of changing the positions of two coupled dimensions of the TES beamline. (Upper right) A quasi-random sample of 16 points from the ground truth. (Lower left) A non-latent GP fitted to the parameter space fitted to the sampled points. (Lower right) A latent GP fitted to the same points, which correctly infers the latent dimensions.

Input parameters for beamlines can be highly coupled, as shown in Fig. 3 ▸. In fitting GPs to beamline data, we adopt a kernel of the form 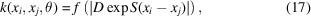
where *f*(*r*) is some radial function, *D* is a diagonal matrix with positive entries, 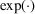 is the matrix exponential and *S* is a skew-symmetric matrix. Because the matrix exponential of a real skew-symmetric matrix is an orthogonal matrix, this kernel represents a norm-preserving transformation in the parameter space by exp(*S*) and a scaling of each dimension in the new basis by *D*. The hyperparameters θ define the entries of *D* and *S*, which for a *d*-dimensional parameter space have *d* and *d*(*d* − 1)/2 degrees of freedom, respectively, together defining a total transformation matrix *T* = *D*exp(*S*) with*d*(*d* + 1)/2 degrees of freedom.

This kernel design is guaranteed to be positive definite so long as *f* is a positive-definite function (from Bochner’s theorem, a function *f* is positive definite if it is the Fourier transform of a weakly positive function on the real line). A commonly used positive-definite function in kernel construction is the Matérn function, which can be written as 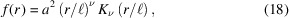
where *K*_ν_(*z*) is the modified Bessel function of the second kind of order ν, and *a*, ℓ, ν > 0 are hyperparameters. For our purposes, ℓ as a lengthscale parameter is redundant and can be subsumed into the hyperparameters defining *T*. This leaves us with a normalized form, 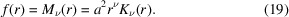
Bessel functions are expensive to compute for arbitrary ν, so we constrain our kernel to ν = 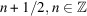, for which equation (19) reduces to the product of a polynomial and an exponential. Unless otherwise specified, we use ν = 5/2 throughout this paper.

### 5.2. Dirichlet-based validity constraints

The application of Bayesian optimization relies on reliable diagnostic feedback, which is often not a realistic assumption for real-life scenarios. Undesirable behavior in the diagnostics can occur both sporadically (*e.g.* in the case of a beam dump or a hardware failure) or systematically (a certain beamline orientation causes the beam to miss a mirror or detector). We want to be able to classify regions of the parameter space as invalid and encode that knowledge into our acquisition function, but we do not want a single unrepresentative glitch to rule out an otherwise worthy part of the parameter space. For this purpose a probabilistic classification model is ideal, and it is also easily able to adjust expectation- and entropy-based acquisition functions.

We use the classification method outlined by Milios *et al.* (2018 ▸) which fits a Dirichlet distribution to the data from which we can generate class probabilities. This method has the benefit of avoiding expensive posterior sampling (as in the case with stochastic variational methods) at the expense of not being able to quantify uncertainty.

A Dirichlet distribution of order *N* is defined as 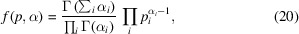
where the vector parameter *p* = {*p*_1_, …, *p*_*N*_} describes the probabilities of classes in an *N* − 1 simplex (so that 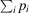 = 1) and the concentration parameters α = {α_1_, …, α_*N*_} parameterize the concentration of the distribution in that simplex. We use transformed GPs to model the concentration parameter α_*i*_ for each classification *i* according to Milios *et al.* (2018 ▸). As the order-*N* Dirichlet distribution is the conjugate prior to the *N*-categorical distribution, we can obtain the probability of a beamline input being valid as 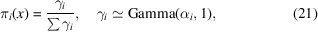
where Gamma(α, β) is the Gamma distribution and γ_*i*_ are samples from that distribution. Using this probability, we can weight any objective-based acquisition function to prefer inputs that lead to valid outputs. This approach has the added benefit of being generalizable to any number of classification labels, which could be made more nuanced than a binary model of validity.

### 5.3. Sampling expense

Bayesian optimization is particularly useful when sampling the objective function *f*(*x*) is expensive. This is strictly true for some beamlines where computing a diagnostic is expensive, *e.g.* those that involve intensive data processing or a complicated meta-routine like a knife-edge scan (Ji *et al.*, 2019 ▸). Many beamlines, though, have no latency in the diagnostics and are only expensive to sample because they are expensive to move around. This is due to the high precision of the motors, which must move slowly so as not to damage the optics, and need time to settle to prevent backlash and make sure that the equipment is exactly at its setpoint; this is typically of the order of several seconds.

A good acquisition function, then, should take into account travel time. A simple solution is to optimize a Monte Carlo acquisition function over a ‘batch’ of points between which we can compute the most efficient route using *e.g. or-tools* (https://github.com/google/or-tools). Ideally, the acquisition function would consider the variable time cost of traveling to a given set of points, but the computational cost of this can be unwieldy.

### 5.4. Hysteresis

Another challenge to machine learning-based optimization is hysteresis, which manifests at beamlines when the actual position of some input varies from the desired input. This can happen when the motor approaches the same position from different directions, primarily from physical backlashes in the hardware. A core assumption of Bayesian optimization is that the relevant function *f*(*x*) always yields the same output (modulo some noise). Hysteresis can be mitigated by overestimating the noise level, or with a more thorough treatment of uncertainty in the inputs of the underlying GP (Liu *et al.*, 2024 ▸). We note the benefit of motor encoders, which can lead to more precise and consistent control of beamline hardware.

### 5.5. Composite objectives

Even though we combine estimates of the different beam attributes into a scalar fitness to be maximized, it is still beneficial to construct and train three separate models for the flux, horizontal spread and vertical spread, a method typically referred to as *composite optimization*. This allows us to take advantage of how different inputs affect different outputs; indeed, many beamlines are designed to separate components that tune the flux from those that tune the focus. This can significantly reduce the effective dimensionality of the alignment problem.

## 6. Implementation

### 6.1. Beamline optimization (*Blop*)

Our beamline alignment tools are implemented in the *Blop* Python package (https://nsls-ii.github.io/blop), relying on the *BoTorch* Python package (Balandat *et al.*, 2020 ▸). In *Blop* we develop a customized kernel which fits to latent beamline dimensions outlined in Section 5.1 and weight common acquisition functions by the probabilistic constraint outlined in Section 5.2. We also use *BoTorch* for model fitting and acquisition function optimization. The algorithm is used in terms of an agent, which we instantiate with motors and diagnostic equipment. We can ‘tell’ the agent about the values of pre-defined objectives (*e.g.* beam height, coherence) and ‘ask’ it for new points to sample. The agent wraps the steps of Bayesian optimization into a single customizable routine [implemented in Python as a .learn() method], which yields a plan accepted by the *Bluesky* experiment orchestration system (in effect, a single button that can be pressed to align the beamline). This routine can be tailored to each beamline (or to each alignment problem for a given beamline). Encapsulating the optimization as a single process simplifies the alignment from the point of view of the user, making experimentation more accessible to users who have less familiarity with a given beamline (*i.e.* hardware, data acquisition, control systems) or with software in general.

### 6.2. Bluesky

*Bluesky* (Allan *et al.*, 2019 ▸; Rakitin *et al.*, 2022 ▸; https://blueskyproject.io/) is a software package that allows for the orchestration and execution of experiments from Python and is in the process of being adapted by various light sources. We have designed *Blop* with *Bluesky* in mind, as it can use *Bluesky* to automatically take data, analyze it and optimize the inputs with the same feedback and control systems used for beamline experiments. This allows the *Blop* agent both to command and control the beamline, leading to an easier implementation, but note that *Blop* is not limited to *Bluesky* facilities and can be made simply to ‘command’ the experiments using only its ‘ask’ and ‘tell’ methods.

*Bluesky* has mainly been developed by NSLS-II, with a growing international collaboration at multiple facilities where it is used and expanded. The adoption of a single standard for experimental control and analysis across many facilities allows us to apply the same automated alignment tools with relatively little effort.

## 7. Experiments

### 7.1. Alignment of a Kirkpatrick–Baez mirror system on the TES beamline

**Figure 4:**
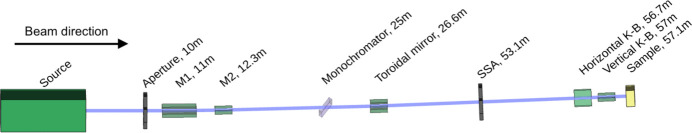
A schematic of the TES (8-BM) beamline at NSLS-II. This representation shows the many optical components that make up modern beamlines, with each optical component having many degrees of freedom that must be optimized in concert in order to carry out experiments effectively.

**Figure 5:**
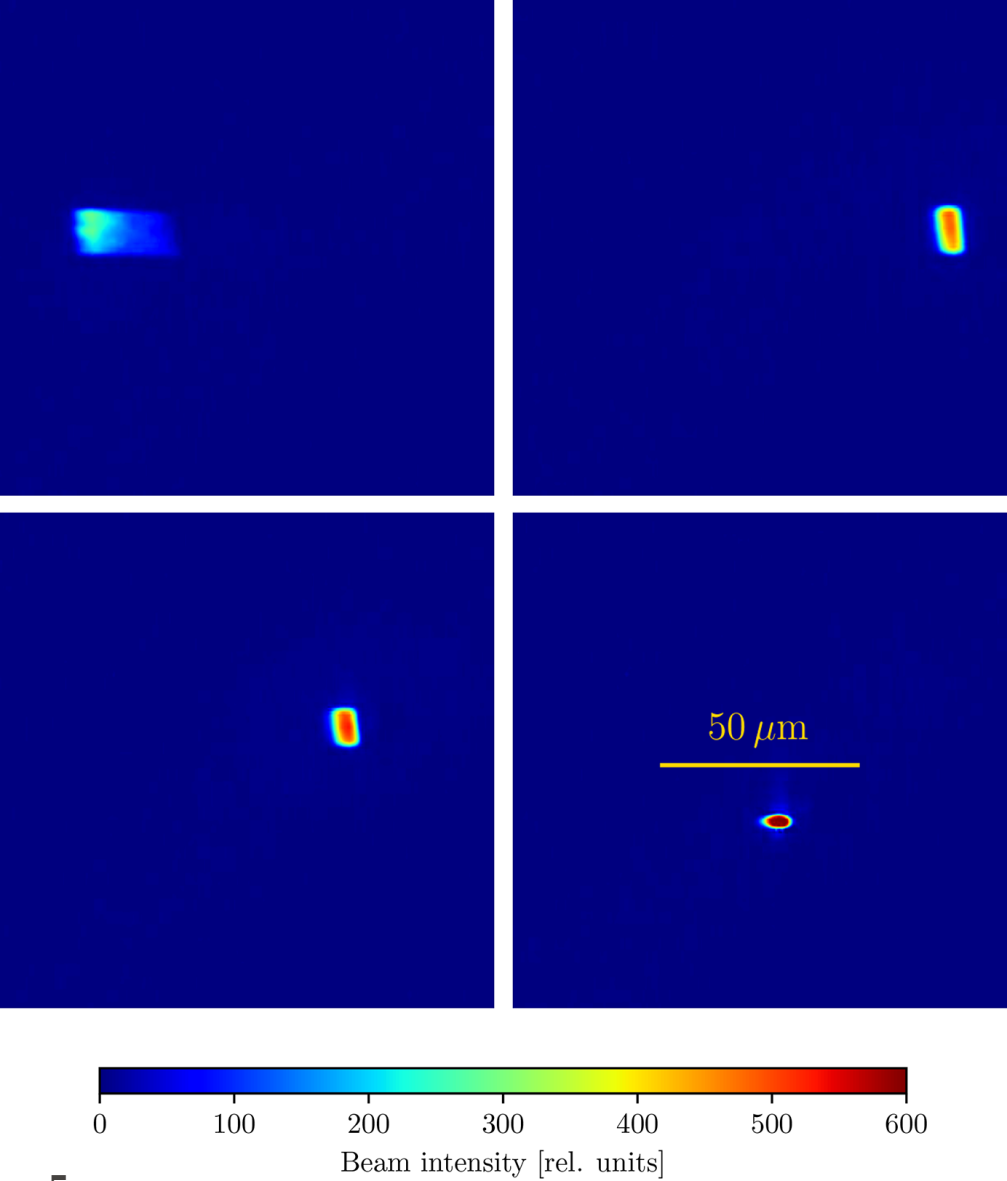
Four different beam configurations on the NSLS-II TES (8-BM) beamline, where the upper left-hand panel shows the initial beam and the lower right represents the optimal alignment. In this alignment test, we adjust the translation and rotation of each of the horizontal and vertical Kirkpatrick–Baez mirrors and the pitch and vertical translation of a toroidal mirror, for a total of six degrees of freedom to maximize the flux density of the beam.

The TES beamline (Northrup, 2019 ▸) is a tender X-ray microspectroscopy beamline at the National Synchrotron Light Source II (NSLS-II) with an energy range of 2–5.5 keV and a beam size which can be tuned between 5 and 20 µm. The X-rays are produced from a bending magnet source and pass through an Si(111) double-crystal monochromator. A toroidal mirror prefocuses the beam onto a secondary source aperture (SSA), after which the beam is refocused onto the sample by a pair of Kirkpatrick–Baez (K-B) mirrors in the endstation chamber. A schematic of the beamline is presented in Fig. 4 ▸. We optimize for the flux density on the sample by allowing each K-B mirror and the toroidal mirror to pitch and translate into and out of the beam for a total of six degrees of freedom. Fig. 5 ▸ shows an example of the beam feedback provided by the camera, with the alignment being gradually improved.

### 7.2. Alignment of a Johann spectrometer on the ISS beamline

**Figure 6:**
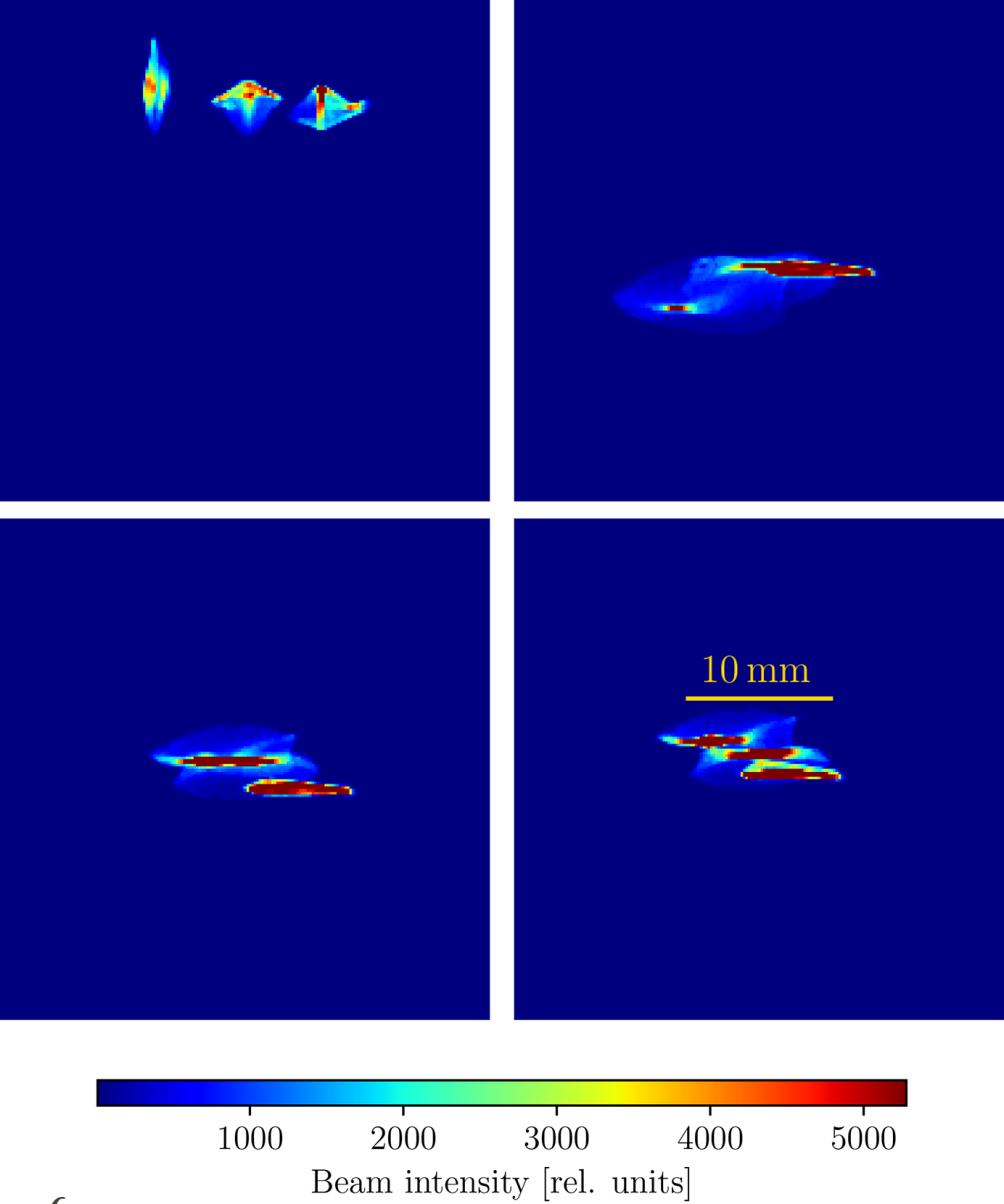
Four different beam configurations on the NSLS-II ISS (8-ID) beamline during automated alignment, where the upper left-hand panel shows the initial beam and the lower right represents the optimal alignment. In this alignment test, we adjust the translation of a central crystal and the translation and pitch of two ancillary crystals for a total of five degrees of freedom to maximize the flux density of the total beam on the area detector.

The Inner Shell Spectroscopy beamline (ISS, 8-ID) beamline (Leshchev *et al.*, 2022 ▸) is designed for X-ray absorption spectroscopy and *operando* and *in situ* characterization of materials. The ISS is a damping wiggler beamline with an Si(111) monochromator capable of producing energies between 4.9 and 33 keV. The beamline is currently developing high-resolution capabilities, with the recent commissioning of a five-analyzer Johann-type spectrometer where, after hitting the sample, the beam is reflected back onto an area detector by several crystals [see Tayal *et al.* (2024 ▸) for an overview of Johann-type spectrometers]. Maximizing the flux on the area detector maximizes the resolution of the spectrometer and so we seek to colocate the reflections of the crystals onto the same point. We use three crystals to focus the beam onto a two-dimensional area detector. Fig. 6 ▸ shows the optimization of the three-crystal system.

### 7.3. Photon transport optimization on the Advanced Light Source beamline 5.3.1

Beamline 5.3.1 at the Advanced Light Source at Lawrence Berkeley National Laboratory is a research and development beamline. It is a bending magnet beamline, operating in the tender X-ray regime (2.4–12 keV photon energy range), where the instrument controls have recently been upgraded to the *EPICS*/*Bluesky* framework.

**Figure 7:**
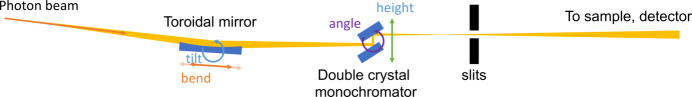
A schematic of beamline 5.3.1 at the Advanced Light Source. The beamline has four degrees of freedom (toroidal mirror pitch and bend, and monochromator angle and height) and four constraints (four-jaw slits).

The photon transport system (Fig. 7 ▸) comprises a first focusing mirror, a monochromator and a few apertures. The focusing mirror is a vertically deflecting toroidal mirror, creating an image of the source at the sample. The mirror is gold-coated with a nominal grazing angle of 5 mrad and mirror-to-object (*p*) and mirror-to-image (*q*) distances of *p* = *q* = 12 m. The corresponding tangential and sagittal radii of curvature are, respectively, *R*_t_ = 2400 m and *R*_s_ = 60 mm. The mirror is bendable along the tangential direction to adjust the vertical focus position. The monochromator is a channel-cut double-crystal Si(111) monochromator providing a 25 mm vertical offset. There are a set of four-jaw slits immediately after the monochromator to block the straight-through beam and another set of four-jaw slits immediately before the sample position (12 m downstream of the toroidal mirror).

**Figure 8:**
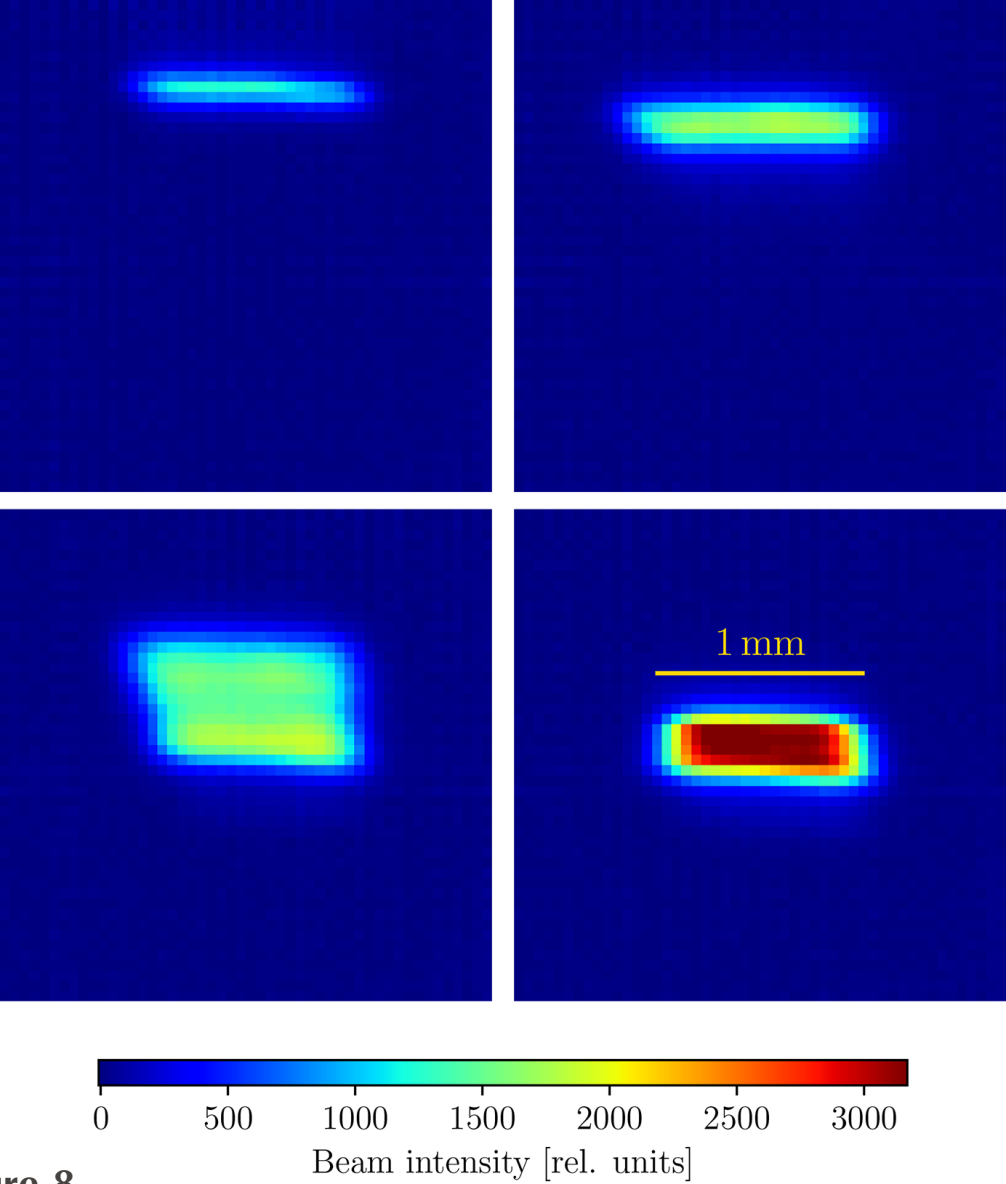
Four different beam configurations on the Advanced Light Source Beamline 5.3.1 during automated alignment. In total, the photon transport has four active degrees of freedom: the focusing mirror pitch and tangential bend, and the channel-cut crystal angle and height. The upper left-hand panel shows the initial manually aligned beam and the lower right the final beam after automated alignment. The upper right and lower left panels show intermediate points collected in the automated alignment process.

For beam measurement, we used a diamond-based X-ray beam monitor (ClearXCam from Advent Diamond) with which we computed the flux as the sum of all pixels. We added a preference for a rounder beam (with some coupling between horizontal and vertical size) by defining an ‘effective area’ metric as
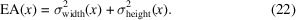
The full scalarized fitness for the effective power density then becomes 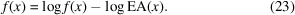
Manual optimization is rendered difficult by the interplay between the toroidal mirror angle, monochromator height and angle, all of them changing the beam height and interfering with the four-jaw slits. Using the described automated alignment, we were able to maximize the power density on the sample in under 5 min, with a final beam size of 1 mm × 0.3 mm (horizontal × vertical, FWHM), close to the theoretical limit calculated by ray tracing (Fig. 8 ▸) and with an improvement in the intensity of more than a factor of two over our best effort using manual alignment.

### 7.4. Alignment of an electron beam at the Accelerator Test Facility

The Accelerator Test Facility (ATF) is a user facility at Brookhaven National Laboratory offering the combination of an 80 MeV electron beam synchronized with a terawatt picosecond carbon dioxide laser (Pogorelsky & Ben-Zvi, 2014 ▸). This gives it the capability to develop cutting-edge electron-beam techniques, including ultrafast electron diffraction and microscopy (McDonald, 1988 ▸), free-electron laser techniques including direct laser acceleration, and using Compton scattering as a high-energy X-ray source (Batchelor *et al.*, 1990 ▸). We modulate three bending quadrupole electromagnets and a solenoid to manipulate the shape of the beam, for a total of four degrees of freedom.

**Figure 9:**
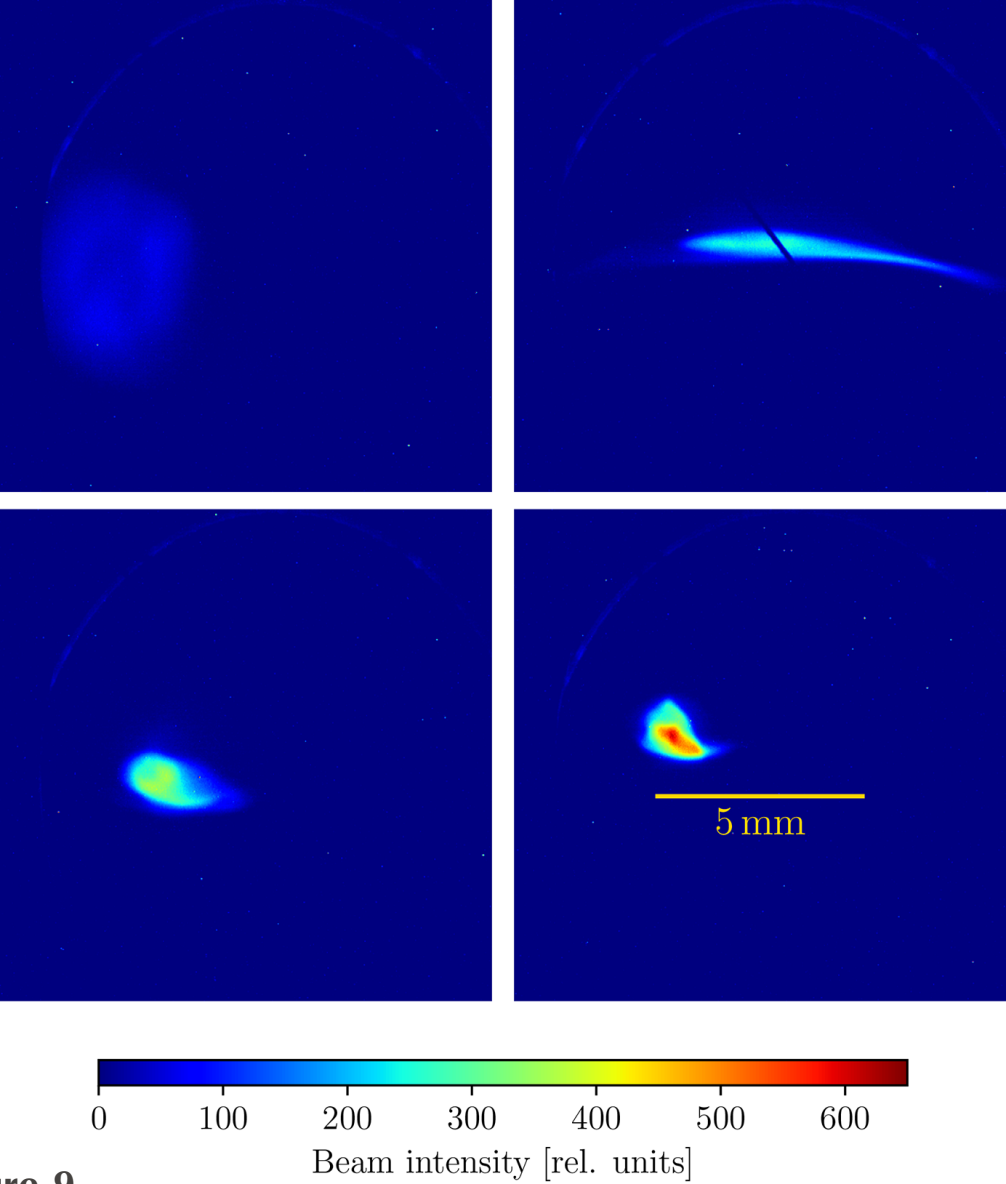
Four different electron beam configurations at the Brookhaven National Laboratory ATF at different stages of automated alignment, where the upper left-hand panel shows the starting beam and the lower right the optimal beam. In this alignment test, we tune the current of four quadrupole electromagnets to maximize the objective in equation (23).

We employ the alternate fitness function in equation (23) which was also used to align the ATF. Fig. 9 ▸ shows an example of the beam feedback provided by the in-house beam diagnostic, with the alignment being gradually improved.

### 7.5. Simulated alignment of the TES beamline

The use of most beamlines is extremely competitive, and benchmarking alignment methods by performing ensembles of different runs is too time-intensive to be viable. Instead, we use digital twins of beamlines using the *Sirepo*–*Bluesky* back end (Rakitin *et al.*, 2023 ▸), allowing us to optimize the beam with the same *Bluesky*-based code used to align real beamlines. We use a ray tracing-based beamline simulation program called *Shadow* (Sanchez del Rio *et al.*, 2011 ▸) to model beam propagation, which does not recreate diffraction effects but accurately recreates the behavior of the beam under mis­alignments. Even this heuristic method is slow, requiring several seconds per scan and thus many hours for comprehensive benchmarking. We note the development of accelerated approximate models of beam propagation under misalignments, which would aid the efficient development of automated alignment tools (Nash *et al.*, 2023 ▸).

**Figure 10:**
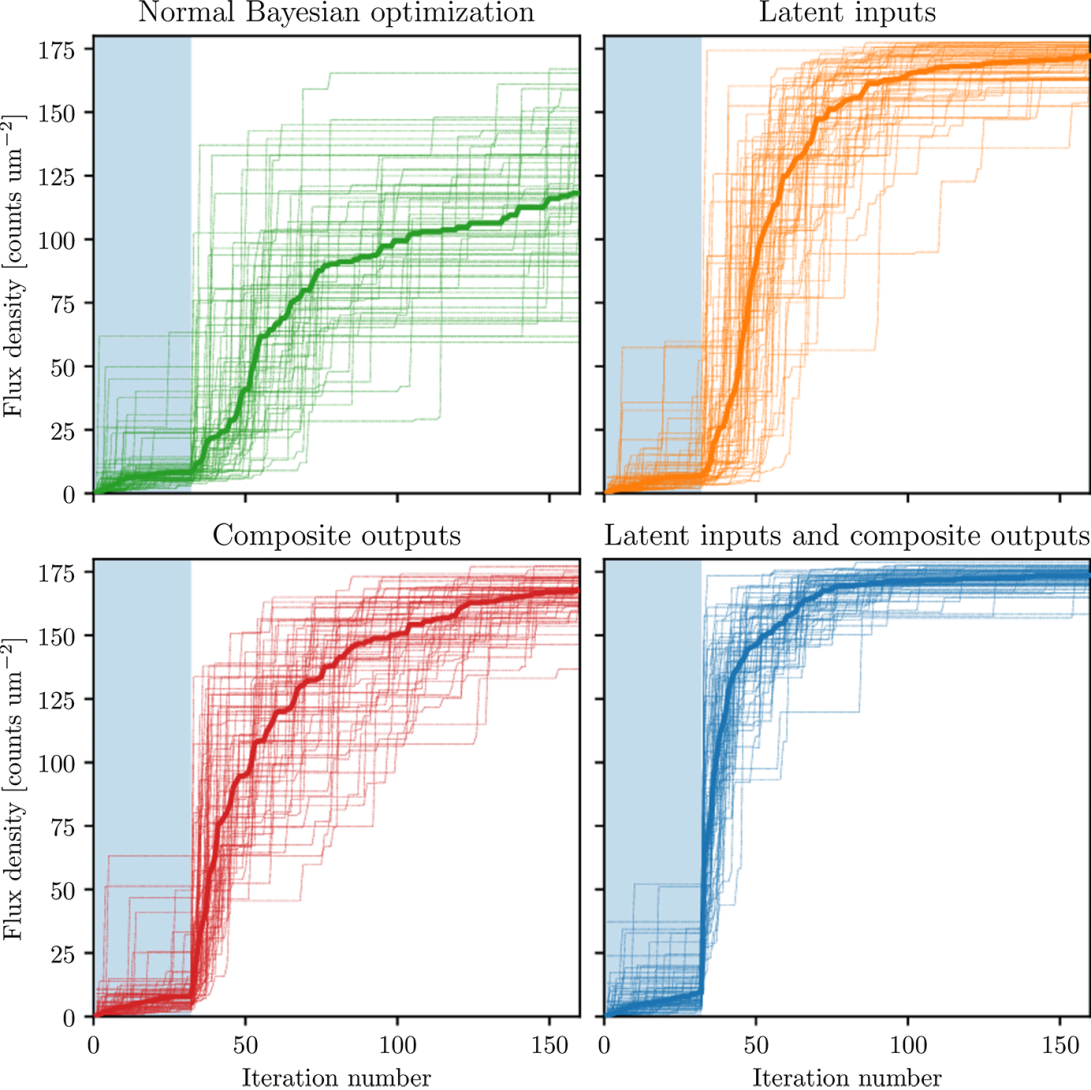
The eight-dimensional optimization of the simulated TES beamline, where the degrees of freedom comprise the toroidal and Kirkpatrick–Baez mirrors. The colors show different varieties of Bayesian optimization algorithms both with and without latent inputs and composite outputs, with both the cumulative maximum of all individual runs (thin lines) and the median cumulative maximum (thick line). Each variety starts out with a quasi-random sampling of 32 points (shaded light blue) and then performs a Bayesian optimization loop with the expected improvement acquisition function. The benefit of using both latent inputs and composite outputs is shown, as we can achieve a better optimum more robustly and more quickly.

For benchmarking, we consider the digital twin of the TES beamline at NSLS-II. In the eight-dimensional case, we use the six degrees of freedom outlined in Section 7.1, but also allow the toroidal mirror to yaw and translate horizontally for a total of eight degrees of freedom. Each K-B motor can move up to ±0.25 mm from a fiducial starting point, while the range of each toroidal motor is bounded by the points where the misalignment of that motor causes the flux through the SSA to fall to 50% of the maximum. The results of this benchmark are shown in Fig. 10 ▸.

**Figure 11:**
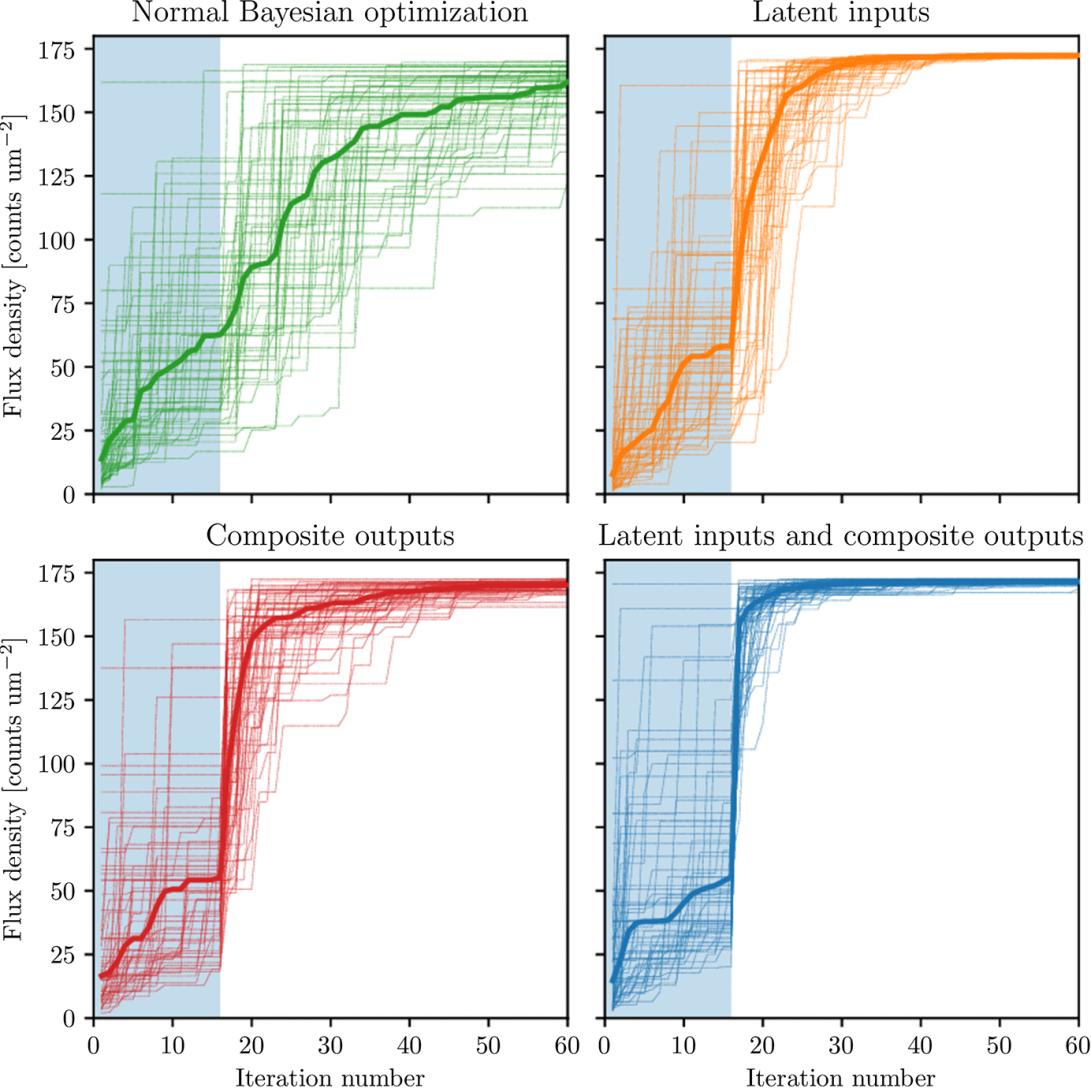
The four-dimensional optimization of just the K-B mirrors, whose motors are each misaligned by up to 0.05 mm. After an initial quasi-random sample of 16 points (shaded light blue), the agent is able almost instantly to return to the optimal alignment.

A simpler benchmark is shown in Fig. 11 ▸, where the agent realigns the four-dimensional K-B system under small mis­alignments (up to 0.05 mm) in each mirror’s motors.

## 8. Further development and discussion

We have applied the same automated alignment tools to several different facilities and have shown that the same Python package can effectively align a range of beamlines. Further refinement of these automated alignment tools will involve applying them to more beamlines at more facilities, with different flavors of optimization problems.

How practical automated alignment can be necessitates an intuitive graphical user interface, from which the configuration of the optimizer is easy to understand. Further development also includes the implementation of new features and better performance in the software. The enabling of Pareto efficient optimization would give the beamline scientist more control over the beam quality, and making the agent take into account the traveling cost of moving the inputs into the acquisition function would allow for more informed optimization. We also plan to allow for a decentralized agent, which can run on a high-performance computing server and communicate with the control system using a streaming system like *Kafka* and feed back to the experiment control using *Bluesky-Queueserver* (https://blueskyproject.io/bluesky-queueserver).

Fly scanning, the strategy of sampling while moving parameters (instead of stopping and settling at each input), presents the potential to speed up beamline alignment, as the sampling expense at most beamlines comes from the accelerating and decelerating of components while varying parameters. This requires a very accurate synchronization between the feedback of inputs and outputs (another use of the motor encoders mentioned in Section 5.4) and is actively being developed at many light source facilities.

We also note that the largest obstacle to applying automated alignment to existing beamlines is the difficulty in constructing robust feedbacks, as many beam diagnostics have non-negligible backgrounds or malfunctioning pixels. While an experienced beamline scientist is able to ignore and look past these artifacts, they may interfere with simpler methods of estimating beam flux, position and size from an image (*e.g.* computing the spread of a profile summed along one dimension). This is especially significant in the case of Bayesian optimization, which relies on accurate sampling of the true objective. This suggests the benefit of more sophisticated diagnostic methods, using machine learning techniques like image segmentation.

## Footnotes

1 In the case of no noise, the support of *p*(*f* ∣ *x*, *y*) consists of only those functions *f* for which *f*(*x*) = *y*.
